# Morphology Evolution of Nanoscale-Thick Au/Pd Bimetallic Films on Silicon Carbide Substrate

**DOI:** 10.3390/mi11040410

**Published:** 2020-04-14

**Authors:** Francesco Ruffino, Maria Censabella, Giovanni Piccitto, Maria Grazia Grimaldi

**Affiliations:** Dipartimento di Fisica e Astronomia “Ettore Majorana”, Università di Catania and MATIS CNR-IMM, via S. Sofia 64, 95123 Catania, Italy; maria.censabella@ct.infn.it (M.C.); giovanni.piccitto@ct.infn.it (G.P.); mariagrazia.grimaldi@ct.infn.it (M.G.G.)

**Keywords:** Au/Pd, SiC, nanomorphology, coalescence, percolation, scanning electron microscopy

## Abstract

Bimetallic Au/Pd nanoscale-thick films were sputter-deposited at room temperature on a silicon carbide (SiC) surface, and the surface-morphology evolution of the films versus thickness was studied with scanning electron microscopy. This study allowed to elucidate the Au/Pd growth mechanism by identifying characteristic growth regimes, and to quantify the characteristic parameters of the growth process. In particular, we observed that the Au/Pd film initially grew as three-dimensional clusters; then, increasing Au/Pd film thickness, film morphology evolved from isolated clusters to partially coalesced wormlike structures, followed by percolation morphology, and, finally, into a continuous rough film. The application of the interrupted coalescence model allowed us to evaluate a critical mean cluster diameter for partial coalescence, and the application of Vincent’s model allowed us to quantify the critical Au/Pd coverage for percolation transition.

## 1. Introduction

Silicon carbide (SiC) is a semiconductor, and ceramic material that has interesting physical and chemical properties that are useful for various applications in different technological areas as a structural material in electronics and optoelectronics [1,2,3]. In these applications, SiC’s characteristic properties, such as a high melting temperature, high thermal conductivity, high Young modulus, wide energy band gap, and chemical stability are fully exploited [1,2,3]. In particular, metal/SiC Schottky diodes are used in the fabrication of different devices, ranging from high-temperature and -power electronics to very sensitive high-temperature hydrogen and hydrocarbon gas detectors and biosensors (due to SiC’s biocompatibility properties) [1,2,3,4,5,6,7,8,9,10,11,12,13,14,15]. So, in the last few decades, to meet specific technological requirements, various methodologies were exploited for the fabrication of metal/SiC diodes. In particular, all te elemental metals and, often, combinations of metals were used to produce SiC-based Ohmic and Schottky contacts with resulting characteristics of barrier height, stability, etc. being widely investigated. In particular, Pd/SiC [1,2,3,4,5,6,7,9,11,13,16] and Au/SiC [1,2,3,4,5,6,16,17,18,19,20,21,22] Schottky contacts with metals in the form of nanoscale-thick deposited films or complex-shape deposited nanostructures attracted much interest for various technological applications ranging from electronics and optoelectronics to sensing and catalysis. In fact, in general, nanoscale-thick metal films and metal nanostructures on functional surfaces are the basis of innovative versatile and high-performance devices [23,24,25,26,27,28,29,30], whose properties are strongly dependent on the morphology, structure, shape, size, and metal–substrate interactions of metal films and nanostructures. Nanoscale metals, in fact, exhibit valuable properties that change by size reduction arising from the electron-confinement effect, and variations of electronic structure and surface effects [23,24,25,26]. These properties are size-, shape-, and composition-dependent, and are exploited in plasmonic, sensing, electrical, and catalytic applications [23,24,25,26]. In addition, regarding nanoscale metals supported on substrates, a specific metal–substrate interaction often leads to an overall composite whose properties arise from the synergistic combination of the properties of individual components, resulting in new functional characteristics. In this regard, nanoscale metals/SiC combinations have potential to reach a high level of efficiency, overcoming the major technical problem related to the low operating temperature of standard metals/Si devices [1,2,3,4,5,6,7,8,9,10,11,12,13,14,15,16,17,18,19,20,21,22]. In particular, nanoscale-thick Pd and Au films or Pd and Au nanostructures fabricated on a SiC surface showed peculiar and interesting properties that are useful, for example, in gas sensing [7,31], biosensing [21,22], and catalysts [32]. Besides pure metals, bimetallic nanoscale metals have shown to have improved characteristics with respect to the characteristics of their individual components [25,33]. In particular, for example, Pd is an excellent catalyst for many reactions with improved effects in nanoscale form [34,35,36,37]. Furthermore, the catalytic activity of nanoscale Pd is often enhanced by the addition of Au [37,38]. In this regard, bimetallic Au/Pd nanoparticles on a SiC surface have recently showed an efficient photocatalytic effect in the hydrogenation of nitroarenes, with a key role played by the catalytic properties of bimetallic Au/Pd nanoparticles and of the specific energy-band diagram of SiC [39]. 

In general, towards real device applications, the critical issue is the controlled direct fabrication of nanoscale metals with the desired structure and morphology on the support substrate. Exploiting physical-vapor deposition approaches for the formation of nanoscale metals on substrates, this can be achieved by controlling the deposition-process parameters once the basic microscopic kinetics and thermodynamics mechanisms, governing the metal growth, are known and related to the growing film’s nanoscale morphology. This control ensures the desired nanomorphology control for specific applications.

On the basis of these considerations, we report on the study of the morphological characteristics of bimetallic Au/Pd nanoscale-thick films sputter-deposited on a SiC substrate. In particular, using the sputter-deposition technique, we deposited bimetallic Au/Pd films on the SiC surface increasing overall film thickness from ~1 to ~10 nm, and we used the scanning-electron-microscopy (SEM) technique to study the surface-morphology evolution of the Au/Pd film versus film thickness. 

The common methods of metal deposition used in SiC device fabrication are sputtering, evaporation, chemical-vapor deposition, and atomic-layer deposition, in order of applicability [40]. However, sputtering is most commonly used today, since it is a fast and economical method that can meet industrial requirements. In addition, metal adhesion is good, and compound targets are possible, as in the case of the Au/Pd target. Evaporation is also common, since it allows higher deposition rates and can be performed in an ultraclean vacuum system. Higher demands are usually placed on the vacuum system, and adhesion can be a problem [40]. Metals can also be deposited by chemical-vapor and atomic-layer deposition, but they are not as common as the other two. An advantage with the chemical-vapor and atomic-layer deposition methods is that epitaxial growth is easier to promote, because of the thermal energy present during the deposition; however, reports on SiC metallization using these techniques are rare [40,41] (instead, for example, atomic-layer deposition is widely used to deposit gate dielectrics on SiC to obtain high-quality interfaces [42]). For these reasons, here we focus on the study of the morphology evolution of the Au/Pd films deposited on SiC substrates by sputtering techniques: results could have a direct connection to industrial technologically important applications.

First, this study allowed us to identify different growth regimes for the Au/Pd film increasing the film thickness: in the initial growth stage (nucleation regime), the nanostructured Au/Pd film was formed by nanoscale dropletlike clusters that evolved into wormlike islands by an interrupted coalescence process, increasing the amount of the deposited metals; further increasing film thickness, a percolation stage was reached for which a discontinuous holed Au/Pd film was obtained; finally, further increasing film thickness, the film became a compact and continuous rough layer. SEM images were quantitatively analyzed to extract the evolution of the mean sizes and surface density of the Au/Pd droplets and islands versus film thickness, and to extract the surface coverage of the Au/Pd film versus film thickness. The plot of the Au/Pd droplets and wormlike-island sizes was analyzed using the interrupted coalescence model to quantify the critical Au/Pd droplet size; after that, the droplets’ nucleation stage ended, and interrupted coalescence growth leading to the formation of the wormlike structures started. The plots of the droplet and island surface density and surface coverage versus film thickness were analyzed by Vincent’s model to quantify the critical Au/Pd surface coverage, after which the percolation stage started. 

These data established a general working framework quantitatively connecting the Au/Pd nanoscale morphology to film thickness, giving the possibility to choose specific deposition conditions to obtain the desired nanoscale morphology of the Au/Pd film for specific applications. These results could be generally useful to improve metallization schemes for SiC, and find applications in some recent technological development, such as in analytical fields related to nanostructure laser desorption/ionization (NALDI) [43,44], and in the epitaxial growth of graphene on SiC with metal intercalation [45,46]. In fact, surface-assisted laser desorption/ionization time-of-flight mass spectrometry using nanoparticles and nanostructured surfaces take the advantages of minimal fragmentation of analytes resulting in extremely high sensitivity. In this regard, nanostructured metal films, as in the case of Au/Pd on SiC, could be very useful to further improve the technique. On the other hand, the application of graphene in electronic devices requires large-scale epitaxial growth. The presence of the substrate, however, worsens charge-carrier mobility. Several works demonstrated the possibility to decouple the partially sp^3^-hybridized first graphitic layer formed on the Si-terminated face of SiC from the substrate by metal (i.e., gold) intercalation, leading to completely sp^2^-hybridized graphene layer with improved electronic properties. A buffer layer on the SiC surface during graphene growth could meet this approach. 

## 2. Materials, Experiment Analysis, and Methods

**Figure 1:**
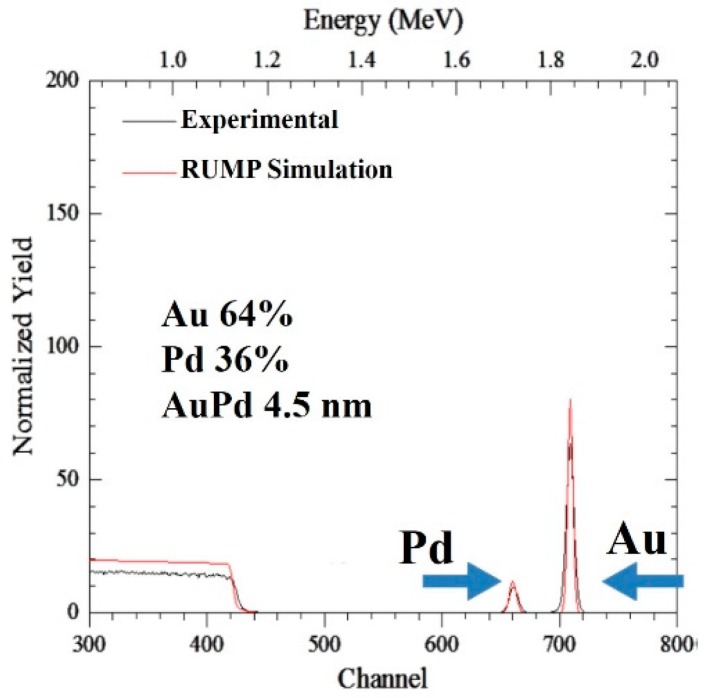
Representative Rutherford backscattering analysis (RBS) spectrum: black line, experimental spectrum; red line, RUMP simulation. Spectrum refers to a Au/Pd film deposited on reference Si substrate. Peaks corresponding to corresponding to presence of Au and Pd are indicated. Simulation of experimental spectrum indicates an atomic composition of 64% Au and 36% Pd film. Correspondingly, an effective thickness for the Au/Pd film of 4.5 nm was evaluated in this case.

The used substrates were 1 × 1 cm n-type 6H–SiC pieces (Si-terminated, doping concentration N_D_ ≈ 5.1 × 10^17^ cm^−3^). The Au/Pd films were deposited on the SiC piece surface by sputter deposition. For these depositions, an Au_60_Pd_40_ (atomic %, nominal composition) target (99.999% purity) was used with a circular shape and diameter of 6 cm. The SiC pieces were located in front of the target at a distance of 4 cm. During the depositions, Ar (0.02 mbar) was used as the working gas. The emission current was fixed to 10 mA (so, the applied power was consequently fixed), and the deposition time was varied to change the effective thickness of the deposited Au/Pd film, the deposition rate being ~1.2 nm/min. However, after the deposition processes, the resulting effective thicknesses of the films were checked by ex situ Rutherford backscattering analysis (RBS) performed on the Au/Pd films deposited on reference Si substrates (to easily interpret the resulting RBS spectra) on which the films were deposited in the same running processes of the SiC substrates. These analyses were performed by using 2 MeV ^4^He^+^ backscattered ions with a scattering angle of 165°. RUMP simulations [47] of the RBS spectra first indicated the effective composition of the deposited films as Au_64_Pd_36_ (atomic %). Then, the RUMP simulations of the RBS spectra indicated the following effective thicknesses for the deposited Au/Pd films: h = 1.2, 2.1, 2.8, 3.5, 4.5, 5.2, 6.9, 8.8, and 10.5 nm with a measurement error of 5%. In particular, RBS analyses allowed to separately evaluate the amount of deposited Au and Pd in atoms/cm^2^ that were converted into Au and Pd thickness by dividing the corresponding measured amount for metal atomic density (5.9 × 10^22^ atoms/cm^3^ in the case of Au; 6.8 × 10^22^ atoms/cm^3^ in the case of Pd) weighted for the corresponding metal atomic percent in the target (0.64 for Au and 0.36 for Pd), and finally summed to give the resulting overall effective thickness of the Au/Pd film. As an example, Figure 1 reports the experimental RBS spectrum (black full line) concerning the Si/Au/Pd where the Au/Pd film resulted in 4.5 nm thickness. The red full line indicates the corresponding simulated spectrum.

Morphological studies were mainly performed with scanning electron microscopy (SEM) employing a Gemini Field Emission Carl Zeiss SUPRA 25 microscope (Carl Zeiss, Oberkochen, Germany). In SEM measurements, a 5 kV accelerated electron beam was used, and a working distance of 3 mm was set. Gatan Digital Micrograph software (Version 3.4.1, Gatan Inc., Ametek, Berwin, PA, USA) was employed to analyze the acquired SEM images; in particular, the brightness of the image was manipulated so to obtain the brighter regions (intensity value set to 1) corresponding to Au/Pd surface areas, and the darker regions (intensity value 0) corresponding to the SiC surface areas. In this way, when identifying particles of a circular cross-section in the SEM image, diameter (width = length = diameter) *R* of the circle was measured for at least 200 particles per sample to construct sizes distributions from which mean diameter <R> and the corresponding error (evaluated as standard deviation) were derived. On the other hand, when identifying islands with an elongated cross-section, the smaller ellipse inscribing the particle for each structure was considered from which width R and length D of the island (ellipse) were measured; this was repeated for at least 200 islands per sample to construct size distributions from which mean width <R>, mean length <D>, and corresponding errors (evaluated as standard deviations) were derived. Surface density *N* (structures/cm^2^) of the Au/Pd particles or islands was evaluated by direct counting and averaging on at least 5 SEM images per sample (and the corresponding error arising from the averaging procedure). Surface coverage P of the Au/Pd film was evaluated as the ratio between the occupied area in an SEM image by the film (bright area) and the total area of the image (bright + dark areas). This procedure was repeated for at least 5 SEM images per sample, and the values of the surface coverage and the corresponding errors were evaluated by the averaging procedure. 

In addition, atomic-force-microscopy (AFM) analyses were carried out by employing a Bruker-Innova microscope (Billerica, MA, USA). The measurements were performed in high-amplitude mode. MSNL-10 Si tips were used (from Bruker) having curvature radius of ∼2 nm. AFM images were analyzed by using SPMLABANALYSES V7.00 software (Version 7, Billerica, MA, USA). AFM images could be similarly analyzed to the SEM images (using, also, line-section profiles of the surface structures) to extract quantitative information on the size of the surface Au/Pd nanostructures, their surface density, and the fraction of the surface area covered by Au/Pd.

In the best setup conditions, the Gemini Field Emission Carl Zeiss SUPRA 25 microscope was in ~1.7 nm lateral resolution [48]. However, in the experiment setup, conditions for SEM measurements in lateral SEM resolution were comparable to those of the AFM measurements (~2 nm, the radius of curvature for the used Si tips) since independent analysis of SEM and AFM images resulted in very similar results for the nanoparticle sizes and surface densities, and for the fraction of surface area covered by Au/Pd. Results obtained by SEM analysis checked by independent AFM measurements are reported below.

## 3. Results and Discussion

**Figure 2:**
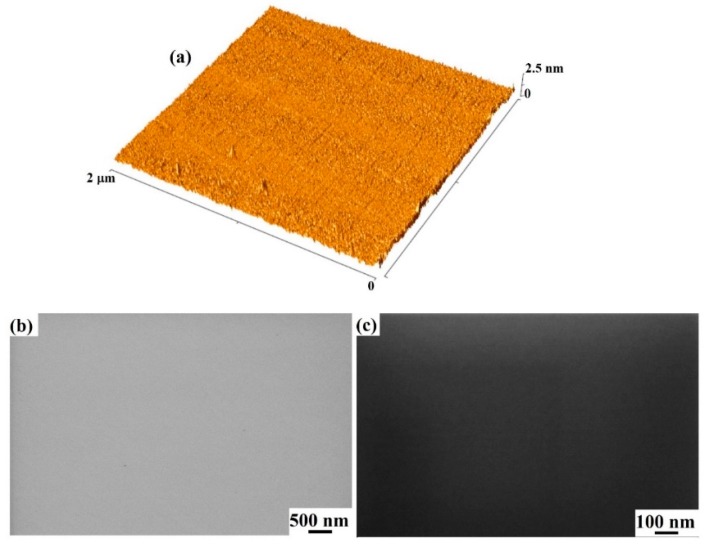
(**a**) A 2 μ × 2 μm AFM image (three-dimensional reconstruction) of starting bare silicon carbide (SiC) surface. (**b**,**c**) SEM images of starting bare SiC surface with magnification increasing from (**b**) to (**c**).

Figure 2 shows the AFM and SEM images of the starting SiC bare surface: (a) 2 × 2 μm AFM image (three-dimensional reconstruction) of SiC surface, (b,c) SEM images of SiC surface (increasing magnification from (a) to (b)). No particular surface features were recognizable either on a large scale (low-magnification image in (a)) or on a small scale (high magnification in (b)). The SiC surface was clean and flat. In this regard, AFM analysis allowed to evaluate that the root-mean square (RMS, i.e., standard deviation of surface heights) equaled to 0.2 nm, confirming the flatness of the starting SiC surface. 

**Figure 3:**
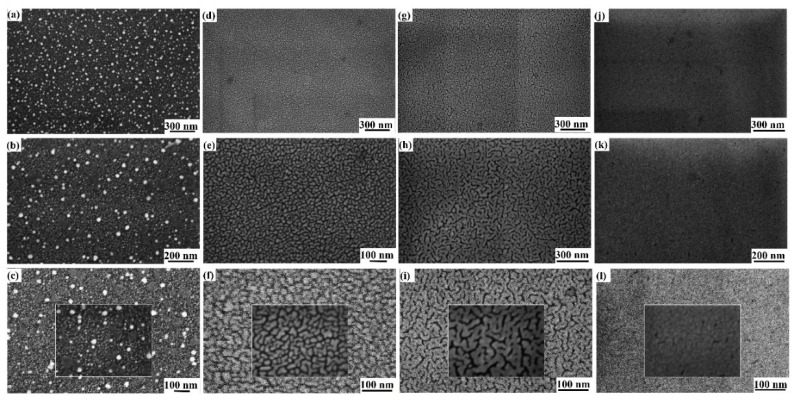
Examples of SEM images of surface of Au/Pd film deposited on SiC surface for different film thicknesses h: (**a**–**c**) h = 1.2 nm and magnification increasing from (**a**) to (**c**); (**d**–**f**) h = 3.5 nm and magnification increasing from (**d**) to (**f**); (**g**–**i**) h = 6.9 nm and magnification increasing from (**g**) to (**i**); (**j**–**l**) h = 10.5 nm and magnification increasing from (**j**) to (**l**). In (**c**), (**f**), (**i**), and (**l**), marker refers to the image in the smaller rectangle where, at parity of the electron-beam scanning rate, a smaller surface area was imaged to reach a better lateral resolution in higher-magnification analysis.

Figure 3 reports the representative SEM images of the surface of the Au/Pd film deposited on the SiC surface for different film thicknesses h: (a–c) h = 1.2 nm and magnification increasing from (a) to (c); (d–f) h = 3.5 nm and magnification increasing from (d) to (f); (g–i) h = 6.9 nm and magnification increasing from (g) to (i); (j–l) h = 10.5 nm and magnification increasing from (j) to (l).

**Figure 4:**
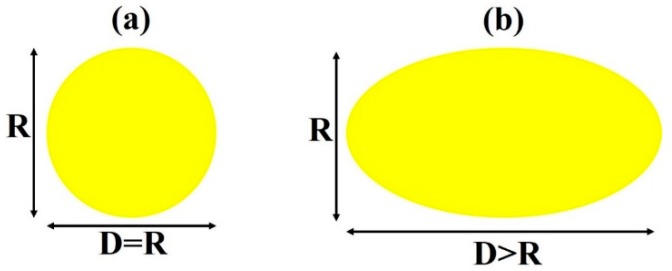
Planar section of an Au/Pd cluster in (**a**) first growth stages (a circle of diameter D) and (**b**) later growth stages (elongated shape represented as an ellipse of characteristic sizes R and D > R).

Concerning the deposited Au/Pd films with thickness h < 3.5 nm, the film is formed by small clusters (nuclei) with a circular cross-section, as can be recognized from the SEM images in Figure 3a–c corresponding to the film with thickness h = 1.2 nm. This is the very first stage of metal growth denoted as the nucleation stage. Metal atoms or small clusters deposited on the substrate surface from the vapor phase are characterized by mobility that leads them to probe a certain surface area and meet other deposited atoms, clusters, or surface defects to start the nucleation process of small three-dimensional islands that grow in size as metal deposition continues [49,50,51]. Both Au and Pd have a strong nonwetting nature on the SiC surface [16,18,52], so starting from the bimetallic Au/Pd target used for the depositions, the Au/Pd film grows in this nucleation regime, following Volmer–Weber growth [51] mode resulting in the formation of a three-dimensional cluster with dropletlike shapes (spherical or hemispherical), with their sizes represented by their diameters R. Increasing Au/Pd film thickness, and in particular for 3.5 ≤ h < 5.2 nm, we observed a transition of the cluster size from the circular cross-sectional shape to an elongated cross shape, as evident from the SEM images in Figure 3d–f corresponding to the film with h = 5.2 nm. In this thickness range, the metal clusters became two-dimensional yet separated islands due to a (partial) coalescence process of the growing metal clusters leading to the formation of the wormlike structures. This shape transition is, roughly, represented in Figure 4, evidencing that, for the elongated metal islands, the two characteristic planar sizes were width R and length D. Such growth evolution is typical for metal films on nonwetting substrates [16,53,54,55,56,57,58,59], described by the interrupted coalescence model (ICM) first proposed by Yu et al. [53]. Starting from the dropletlike metal clusters obtained in the nucleation stage, further increasing the amount of deposited metal atoms, the growing clusters can touch each other, giving origin to a coalescence process (two or more clusters merge to form a unique cluster with volume equal to the sum of the volumes of the contacting clusters, but with a lower surface area than the sum of the surface areas of the contacting clusters) to minimize the total surface area of the system. Therefore, at this stage, the thermodynamic change drives system transformation towards the minimum of surface energy. However, in this coalescence process, the substrate can act by a wiping action leading to a partial coalescence process in which part of the substrate, which was initially covered by metal, is then wiped clean. As a result, the metal film is formed by elongated islands resulting from full coalescence in one direction and partial coalescence in another direction. The islands are separated by gaps between them. The ICM model identified critical diameter R_c_ for the dropletlike growing metal clusters separating the nucleation and growth stages to partial (interrupted) coalescence, being R_c_-dependent on the chemical nature of the metal and substrate, and on temperature. 

Further increasing the amount of deposited metals with respect to the partial-coalescence stage, a percolation regime occurs, in our case, for Au/Pd film thickness of 5.2 < h ≤ 8.8 nm. Percolative morphology for the Au/Pd film can be observed in the SEM images in Figure 3g–i corresponding to the film with h = 6.9 nm. In this growth regime, the increased amount of deposited metal led to the continuous growth of the two-dimensional coalesced islands, which grew much longer than larger until connecting with each other to form a quasicontinuous (holed) network across the surface. Finally, further increasing the amount of deposited metal, a compact, rough, continuous film was obtained by a filling process of the holes that were present in the previously obtained percolative film. In our case, this occurred for 8.8 < h ≤ 10.5 nm. As an example, the continuous, rough Au/Pd film can be recognized in the SEM images in Figure 3j–l corresponding to the film with h = 10.5 nm.

**Figure 5:**
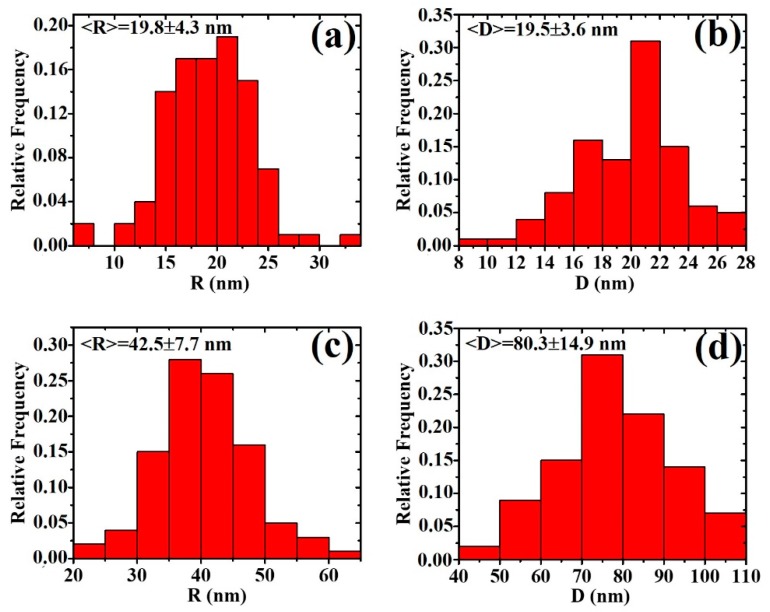
Representative size distributions for Au/Pd clusters: (**a**,**b**) distributions for width R and length D, respectively, of Au/Pd clusters in deposited film of h = 2.8 nm thickness; (**c**,**d**) distributions for width R and length D, respectively, of Au/Pd clusters in deposited film of h = 5.2 nm thickness. In each case, mean values of width <R> and length <D>, as extracted by the distribution, are reported.

**Figure 6:**
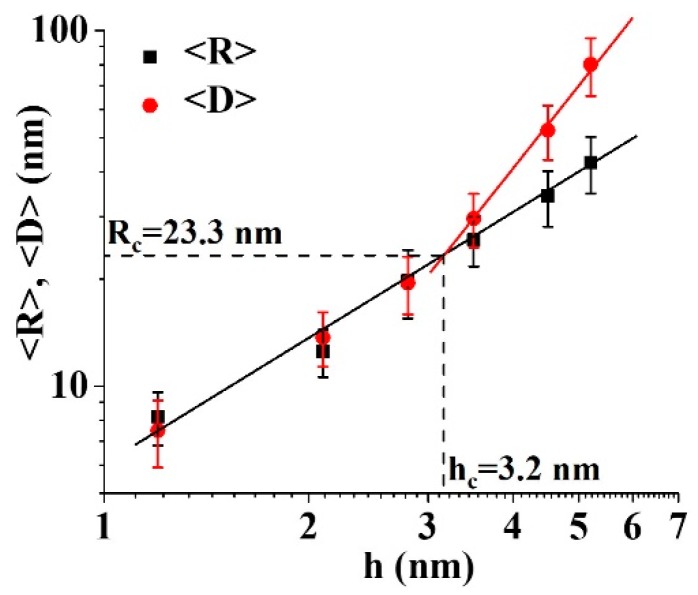
Evolution plot of mean width <R> and mean length <D> of Au/Pd islands versus thickness h of the deposited Au/Pd film, for 1.2 ≤ h ≤ 5.2 nm. Full lines arose by fitting procedures of experiment data.

We can characterize the partial-coalescence and percolation stages by the quantitative evaluation and analysis of some parameters. First, we used the SEM images (see Section 2) to derive the values for the mean sizes of the Au/Pd surface structures versus film thickness. In the nucleation stage, we evaluated diameter R of the circular cross-section of the dropletlike Au/Pd particles to construct diameter distributions from which mean diameter <R> (and the corresponding standard deviation) was extracted. As an example, Figure 5a,b reports, respectively, the distribution of width R and length D for the dropletlike particles observed in the sample with an Au/Pd film thickness of h = 2.8 nm; <R> = <D> ≈ 19.5 nm, confirming the circular cross-section of the Au/Pd structures characterized by unique mean diameter <R>. In addition, in the partial-coalescence stage, we evaluated width R and length D of the partially coalesced wormlike Au/Pd structures to construct the distributions for R and D, from which the mean values <R> and <D> (and the corresponding standard deviations) were extracted. An example is shown in Figure 5c,d, where the distributions for R and D corresponding to the Au/Pd structures were observed in the Au/Pd film with thickness of h = 5.2 nm. In this case, <R> = 42.5 nm and <D> = 80.3 nm, confirming that, in the partial-coalescence stage, (<D>) > (<R>), a condition characteristic of elongated Au/Pd structures. Overall, Figure 6 reports (on a log–log scale) the experimentally derived values (dots) of mean island width <R> and length <D> as a function of film thickness h (for 1.2 ≤ h ≤ 5.2 nm). For h = 1.2, 2.1, and 2.8 nm, condition <R> ≈ <D> was realized; for h = 3.5, 4.5, and 5.2 nm, condition (<D>) > (<R>). In the log–log scale of the plot in Figure 6, data <R> versus h and <D> versus h were fitted by linear functions (black full line for <R> versus h with 1.2 ≤ h ≤ 5.2 nm, red full line for <D> versus h with 3.5 ≤ h ≤ 5.2 nm). The intersection of the two full lines allowed to evaluate critical radius R_c_ defined by the ICM model from which the partial-coalescence process of the Au/Pd clusters began.

So, we obtained R_c_ = 23.3 nm corresponding to the critical thickness for the Au/Pd film h_c_ = 3.2 nm. Therefore, at critical thickness 3.2 nm of the deposited Au/Pd film (corresponding to a mean critical Au/Pd cluster diameter of 23.3 nm), the nucleation and growth stages of the dropletlike Au/Pd clusters ended, and the partial-coalescence stage started. Continuing the metal deposition, a later stage of the Au/Pd film growth occurred: the elongated islands grew larger and longer, and when the peripheries of the neighboring islands met and touched, the onset for percolation occurred. To characterize the percolation-growth stage, we used Vincent’s model [59,60,61]: in this model, the transition from isolated islands to percolation occurs at critical film-percolation coverage *P_c_*. In the framework of Vincent’s model, film-percolation coverage *P* is related to metal-island surface density *N* by (in the hypothesis of noninstantaneous coalescence [61])

(1) $$P=P_{0}\left(N/N_{0}\right)+P_{c}\left[1-\left(N/N_{0}\right)\right],$$

where *P*_0_, initial metal coverage; *N*_0_, initial metal-island surface density (*N* = *N*_0_ when *P* = *P*_0_); and *P_c_*, critical film coverage for which percolation occurs. The application of Equation (1) requires to know *N*_0_. In this regard, in the framework of Vincent’s model, the metal-island surface density was dependent on film thickness by [59]

(2) $$\ln\left(N/N_{0}\right)=-Ah^{2/3},$$

where *A* is a constant determined by the shape of the islands.

**Figure 7:**
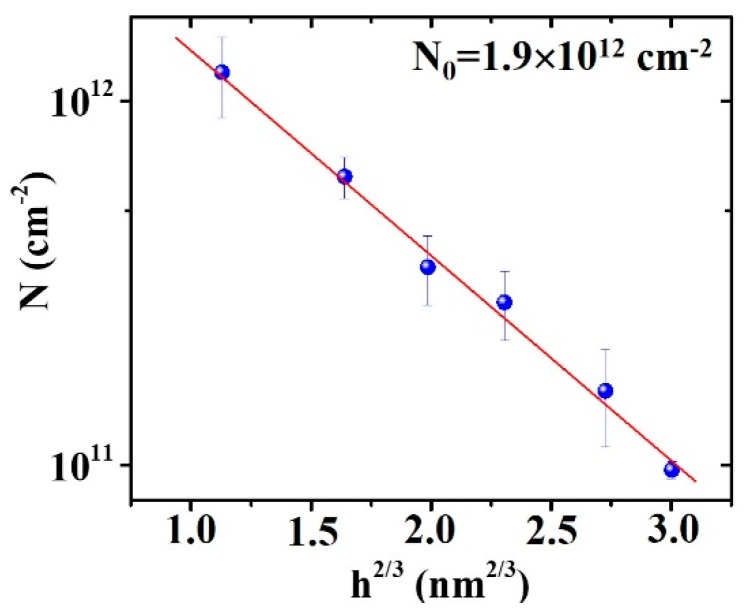
Plot of mean number of Au/Pd islands (LogN) versus *h*^2/3^ with h being the thickness of the deposited Au/Pd film (with 1.2 ≤ h ≤ 5.2 nm). Full line arose by the fitting procedure of the experiment data.

On the basis of these theoretical results, after evaluating the mean values of surface density N for the Au/Pd clusters and islands for 1.2 ≤ h ≤ 5.2 nm, Figure 7 reports, on a semilog scale, the values of *N* (dots) versus *h*^2/3^, so that, from the linear fit of the experiment data, value *N*_0_ = 1.9 × 10^12^ cm^−2^ (considered as fitting parameter) was obtained for the initial surface density of the Au/Pd clusters. 

**Figure 8:**
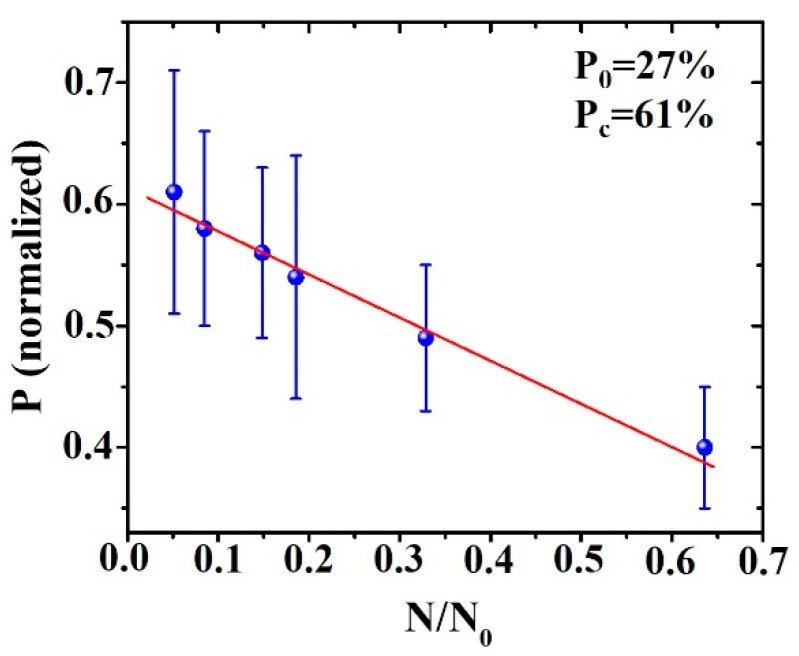
Plot of fraction of surface area covered by Au/Pd film *P* versus number *N* of Au/Pd islands normalized to *N*_0_ (starting island density).

Once *N*_0_ was evaluated, we reported the experiment-derived values of Au/Pd surface coverage *P* (evaluated for each film thickness h) versus *N*/*N*_0_ (with N the Au/Pd island surface density evaluated for each corresponding film thickness h); see the dots in the plot in Figure 8. The linear fit of the experiment data (full line in Figure 8) allowed to evaluate the value of the initial Au/Pd surface coverage as fitting parameters, *P*_0_ = 27%, and, in particular, the critical value for Au/Pd surface coverage at which the percolation stage for the Au/Pd film occurred, *P_c_* = 61%.

## 4. Conclusions

We studied the growth morphology of nanoscale-thick (1–10 nm) bimetallic Au/Pd film that was sputter-deposited, at room temperature, on 6H–SiC, versus film thickness h. Increasing film thickness, we observed the evolution of the Au/Pd film as formed in the initial stage of growth (h < 3.5 nm, nucleation regime) by three-dimensional isolated particles with circular cross-section to a film as formed by partially coalesced wormlike islands (3.5 ≤ h < 5.2 nm, partial-coalescence stage), to a percolative film (5.2 < h ≤ 8.8 nm, percolation stage), and finally to a continuous, compact, rough film (h = 10.5 nm). The interrupted-coalescence model was applied to describe this growth sequence, allowing to estimate critical mean Au/Pd particle diameter *R_c_* = 23.3 nm (corresponding to critical film thickness h_c_ = 3.2 nm) to start the partial-coalescence regime. Furthermore, the evolution of the Au/Pd particle surface density and coverage *P* was analyzed by using Vincent’s model to analyze, in particular, the percolation process at the later stage of growth, allowing to evaluate percolation threshold *P_c_* = 61%.

These results set a general framework that can connect Au/Pd nanoscale morphology to film thickness, giving the possibility to controllably tune film morphology for specific miniaturized-device applications.
